# Validation of GPS-Based Monitoring and Remote Sensing of Ice-Shelf and Ice-Sheet Movement Changes

**DOI:** 10.3390/s21237822

**Published:** 2021-11-24

**Authors:** Xiuhong Li, Xuejie Hao, Lizeyan Yin, Lu Liu, Yushuang Ma, Rongjin Yang, Qiao Song

**Affiliations:** 1 State Key Laboratory of Remote Sensing Science, College of Global Change and Earth System Science, Beijing Normal University, No.19, Xinjiekou Wai Street, Haidian District, Beijing 100875, China; lixh@bnu.edu.cn (X.L.); haoxuejie@mail.bnu.edu.cn (X.H.); liulubnu033@mail.bnu.edu.cn (L.L.); mays@mail.bnu.edu.cn (Y.M.); 201621490041@mail.bnu.edu.cn (Q.S.); 2 Institute of Computing, Modeling and Their Applications, ISIMA, University Clermont Auvergne, 63000 Clermont Auvergne, France; Lizeyan.YIN@etu.uca.fr; 3 Chinese Research Academy of Environmental Sciences, No.8, Beijing 100012, China

**Keywords:** GPS, ice shelf and ice sheet movement, monitoring system, remote sensing validation

## Abstract

The north and south poles of the earth (hereinafter referred to as the polar regions) are important components of the earth system. Changes in the material balance and movement of the polar ice shelf reflect the influence of the polar regions on global climate change and are also a response to global climate change. Through a comprehensive investigation of ice-shelf kinematics, with sufficient accuracy, it is possible to obtain ice-shelf elevation, movement-state data, ice-shelf material balance state, and the ice-shelf movement dynamics mechanism. Due to the extremely harsh environment in polar regions, remote sensing is currently widely used. Manual and equipment monitoring methods show insufficient accuracy or discontinuous time series. There is an urgent need to obtain continuous real-time ice-shelf kinematics-related parameters on the ground to verify the reliability of the parameters obtained by satellite remote sensing. These parameters should be combined with remote sensing monitoring to provide data support. In this paper, a monitoring system for the movement of polar ice and shelf ice cover is developed, and it is proposed that various data can be acquired by integrating high-precision GPS (global positioning system) and other sensors. Solutions to the problem of low-temperature power supply in the polar regions, data acquisition and storage strategies, and remote communication methods are proposed. Testing and remote sensing validation verified that the developed acquisition system can fulfill the requirements for monitoring the movement of the polar unmanned ice shelves and ice sheets.

## 1. Introduction

The north and south poles of the earth (hereafter referred to as the polar region) are important parts of the earth system, are drivers of climate change, and are cold sources of global climate change [1]. These poles are important driving factors of sea-level increases in the context of global warming. Polar research plays a key role in global change, especially in global climate change research. The ice shelf, an important part of the polar region, refers to a large area of fixed ice floes at sea connected to the continental ice. Glaciers are the source of the ice shelf, and the ice shelf is the source of the icebergs. The changes in polar ice shelves reflect both the impact of polar regions on global climate change and the response of polar regions to global climate change [2]. Through a comprehensive investigation of the kinematics of the ice shelf and information on the surface material balance, the elevation changes and motion state of the ice shelf can be obtained. This information can be used to study the ice-shelf material-balance state and the dynamic mechanism of ice-shelf movement, as well as to reveal the impact of climate change on the ice-shelf material balance and motion state [3,4].

The purpose of the kinematic study of the ice shelf and ice sheet is to determine how the ice shelf moves, how the movement of the ice shelf changes and how the movement of the ice shelf is affected by other factors. With the advent of satellite remote sensing technology and new measuring technology, more and larger-scale parameters related to ice-shelf kinematics have been obtained, including the grounding line of the ice shelf, ice flow velocity, strain rate, ice elevation, ice-shelf thickness and width, net surface accumulation rate, bottom freeze-thaw rate, and tidal information related to vertical height changes of the ice shelf [5].

The ground measurements of the ice shelf and ice sheet mainly contain conventional ground measurements, GPS (global positioning system) monitoring and airborne ice radar data, etc., among which GPS monitoring is the most widely used. Specifically, conventional ground measurements can obtain data on the snow accumulation rate, ice flow rate, and strain rate, while airborne ice radar measures the ice thickness. The ice kinetic parameters obtained with the aid of GPS include the ice flow velocity, strain rate, surface elevation, slope, and tidal information. Additionally, seismometers can measure changes in the front end of the ice shelf. Currently, GPS monitoring technology is a mature technology to monitor the change in glaciers and ice shelves [6]. The 19th Antarctic expedition team (2002–2003) established a GPS observation site on the Amery ice shelf. Through five consecutive days of observation, the tidal changes at the edge of the ice shelf and the information on the ice-shelf flow rate were obtained through joint measurement with the Zhongshan Station of China and the GPS base station in Australia [7]. However, operation of the abovementioned GPS monitoring means requires professionals on site. Even in emerging unattended GPS tracking stations, the received data are stored in the instrument’s memory and are then retrieved by a person after a certain period. Therefore, it is necessary to develop an unattended automatic GPS monitoring system for the harsh environment of the polar region that can transmit data periodically.

Janssen et al. (2002) developed an unattended GPS single-frequency monitoring system for deformation monitoring of the Papandayan volcano in Indonesia. Powered by solar panels and rechargeable batteries, this system can realize real-time transmission of observation data through radio technologies. More and more GPS devices are used for bridge deformation monitoring [8]. Similar technologies have been successfully applied in the monitoring of the polar region. Motyzhev et al. (2017), at the Marine Hydrophysical Institute of RAS, developed the BTC60/GPS/ice temperature-profiling drifters for investigation of polar areas [9]. The drifters could measure air pressure, water temperatures, ocean pressures and current locations. Siegfried et al. (2017) used GPS interferometric reflectometer (GPS-IR) technology to measure the thickness changes of fir pillars (<2 m) on the 23-station GPS array in western Antarctica. GPS-IR is an effective technology to monitor the process of surface mass balance, which can be applied to historical GPS data sets and future experiments to provide key in situ observations of the process of driving surface-height evolution [10].

Airborne and satellite remote sensing monitoring of ice shelves mainly includes the application of optical images, such as the Landsat series and MODIS (moderate-resolution imaging spectroradiometer), to determine the ice-shelf grounding line, ice-shelf width, and iceberg disintegration and acquire the ice-shelf surface-ice flow velocity based on multi-temporal tracking [11,12], using satellite altimeters, such as Seasat, Geosat, ERS-1/2, Icesat, etc. Algebraic elevations are obtained using radar satellites, such as Radarsat-1/2 interferometric radar, to generate topographic maps and determine ice flow rates and strain rates; ice-shelf collapse is determined using gravity satellites, such as Grace, to measure changes in the ice-shelf material distribution [13]. The large-area instantaneous imaging characteristics of remote sensing technology have greatly promoted the study of ice-shelf kinematics, but the reliability of the parameters obtained by satellite remote sensing still needs validation using observations from ground stations. In addition, there is a tremendous increase in the number of satellites currently available for polar monitoring, making the acquisition of long-term sequence data possible. Because of the lack of high-frequency monitoring data, the daily and monthly variation of ice-shelf movement, the transient response of emergencies, and the long-term sequence of tidal information related to the ice shelf cannot be observed, so many ices-shelf changes still cannot be explained reasonably. In particular, satellites usually acquire data on the disintegration of ice shelves and other abrupt events after their occurrence, so the specific time of the change and changes in the ice-shelf motion parameters at the time of occurrence cannot be accurately obtained.

Thus, the development of an ice-shelf GPS continuous automatic-monitoring system based on wireless-sensor-network technology (hereafter referred to as the system) can provide data support for relevant scientific research. The system can obtain continuous measured data on the ground and combine it with multi-source satellite remote sensing data of large-area instantaneous observations [14].

## 2. System Design

### 2.1. Overall Frame Diagram of the System

**Figure 1:**
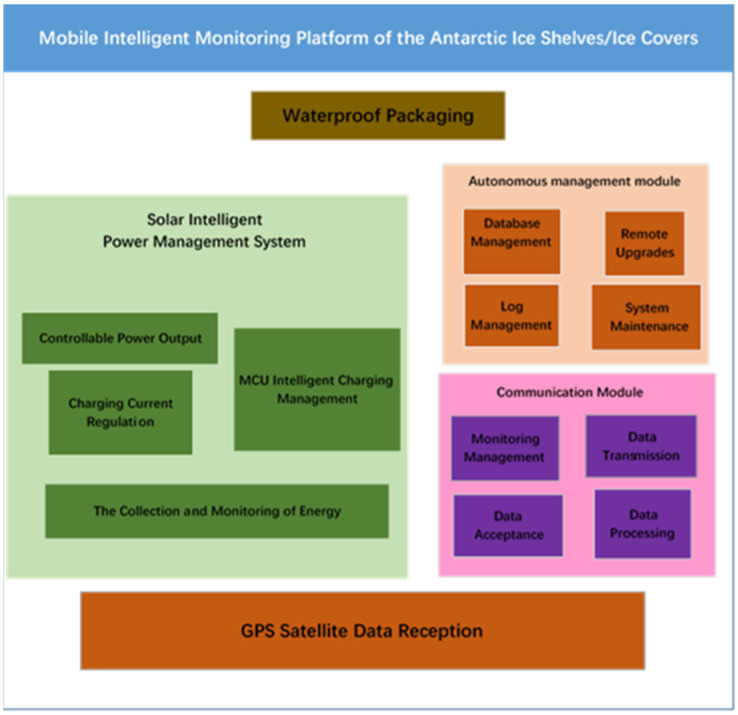
System structure.

To operate normally and continually in the extreme environment of Antarctica, the system is required not only to resist extremely low temperatures and other extreme conditions but also to have intelligent management control capabilities because it is very difficult for humans to reach certain monitoring points. Intelligent structural design can greatly reduce human operations [15,16]. In addition, energy consumption in the Antarctic is larger than that in other non-polar regions. Thus, the system is divided into five modules: GPS satellite-data-receiving module, autonomous management module, communication module, power-management module, and waterproof thermal-insulation package. Its structure is shown in Figure 1.

The power-management module provides energy for other modules and is the basis of monitoring equipment. The autonomous management module performs tasks such as adaptive scheduling, code upgrade, and fault detection of the monitoring equipment, which is the key to the normal operation of the monitoring equipment under unmanned management. The GPS satellite-data-receiving module is the core module of the monitoring equipment and obtains the location-change data of the monitoring equipment. The communication module transmits the data obtained by the monitoring equipment to the receiving data-terminal equipment after preprocessing, which is an important part of the testing equipment. The waterproof and heat-preserving packaging module provides a good operating environment for the ensemble of monitoring equipment and ensures the normal operation of other modules under extremely harsh conditions. The five modules that comprise the monitoring equipment cooperate with each other in the scheduling and control of the embedded operating system and jointly ensure the normal operation of the equipment.

The GPS satellite-data-receiving module mainly includes two parts: a dual-frequency static high-precision GNSS (global navigation satellite system) receiver and data transmitter. The core board of the system can control the opening time of the GNSS receiver and transmit the data to the main board through the RS232 serial port USART (universal synchronous/asynchronous receiver/transmitter) protocol. The main board then stores the data in the local SD card (secure digital memory card) and sends it remotely.

The autonomous management module can complete the scheduling of request tasks, remote code upgrades, system fault detections, and remote execution of monitoring tasks under unattended conditions.

The communication module includes multiple interfaces (serial ports, 485 bus interfaces, etc.) of the acquisition and transmission module, as well as an energy-level estimation device. Dynamic scheduling of communication tasks by energy-saving strategies can achieve the aggregation and unified service of communication tasks to reduce communication energy consumption.

The power management module coordinates the solar-energy acquisition and energy-consumption control of hardware through an energy-management control strategy library. Dynamically controlling energy consumption based on current task operations will minimize energy consumption.

The special waterproof thermal-insulation package is designed to ensure that the equipment operates normally under the extreme environmental conditions of the Antarctic, such as low temperature and heavy snow.

The system was developed and customized for the special environment of Antarctica, which can meet the requirements of work in extreme environments, such as low temperature, strong wind, and extreme day and night. In addition, it has certain advantages in the following aspects: (1) Independent research and development of the embedded operating system, BNUOS. Under its unified management and scheduling, the GPS receiver equipment is filled with insulation materials to ensure the normal operation of the instrument under extremely low-temperature conditions. Remote-control technology is adopted for energy to realize unattended long-term continuous monitoring. (2) The acquisition of continuous, high-sampling frequency, high-precision, long-term series of polar-ice-shelf motion data sets enables the study of the ice-shelf-system status on a smaller time scale. (3) Developed automatic data preprocessing technology to automatically compress the acquired data and improve the efficiency of data transmission.

### 2.2. Hardware Design

After consideration of the data needs, the energy supply required for polar monitoring, the extremely cold environment in Antarctica, and other factors, the hardware of the system consists of five parts: core processor, data storage, GPS data collection, intelligent solar-energy power-management system, and communication.

#### 2.2.1. The Core Processor

The core processor is the core part of the system, which controls the operation of the entire system, including time management, task deployment, data acquisition, storage and communication, power management, and other important tasks. Its components are mainly microprocessors, peripheral circuits, and clock management. 

To meet the requirements of low power consumption, ST System’s 32-bit ARM STM32F107 was selected as the main control chip. The peripheral circuits include a reset circuit and a JTAG (joint test action group) circuit, which is mainly used for software programming and resetting of the chip. To ensure the normal operation of the whole system under low-temperature conditions and a more accurate time system of the processor, an external 12 MHz temperature-compensated crystal oscillator was used to provide a basic clock source for the chip.

#### 2.2.2. Data Storage

Due to the extreme environment of Antarctica, remote communication is very difficult. For example, communication interruption or data loss may occur during remote communication. Therefore, to enable the system to retain the original data when there is a problem in the system’s remote-communication data transmission, the system uses an external SD card to store the backup data. The SD card and the processor adopt the SPI protocol communication principle to store and read data.

#### 2.2.3. GPS Data Collection

This part uses a dual-frequency static high-precision GNSS receiver to receive the satellite signals. The receiver has many advantages, such as full-constellation and full-band satellite reception, a fully integrated design that makes it easy to carry and use, and polymer sealing technology that guarantees that the system works normally under extreme conditions, which all indicates that the receiver is suitable for GPS measurements under the extreme conditions of the Antarctic. The receiver’s nominal horizontal accuracy during static measurements is ±(2.5 + 1 × 10 − 6 × D) mm (D is the baseline length), and the vertical accuracy is ±(5 + 1 × 10 − 6 × D) mm. Moreover, the system’s operating power consumption is only 2.5 W, which greatly reduces energy consumption.

The receiver is connected to the mainboard circuit through the RS232 interface for full-duplex communication. When the system is running, the mainboard sends a serial port command to the receiver through the serial port, and the receiver transmits the original observation data or ephemeris data to the mainboard according to different instructions. The data mainboard stores the data directly and sends the data remotely to the receiving server when the remote communication task is turned on.

#### 2.2.4. Intelligent Solar Energy Power-Management System

The energy acquisition method that is required to power a system in a polar region is an uphill task, especially when the system is designed to automatically acquire and remotely transmit GPS data unattended, which means that energy acquisition is critical to the operation of the system. To ensure that the unattended automatic operation system uses solar energy as the energy source of the system for many years, including during the extremely long nighttime, the system will reduce the observation frequency according to the electric quantity. This part has been applied in other developed environmental parameter-monitoring systems in polar regions [17], which have obtained a large amount of data after nearly three years of movement of the Antarctic ice sheet.

#### 2.2.5. Communication

The communication system completes the acquisition and transmission of GPS signals under unattended conditions. The means of remote communication will use wireless microwave communication or China Unicom SMS (Short Message Service). The wireless microwave will convert the USART protocol data to UDP (User Datagram Protocol) protocol data through a data-transparent transmission module of RS232 to the Ethernet interface. Then, the communication module packs the data into short messages and transmits the data to the Zhongshan Station server by microwave communication. Afterward, the data are sent to the mainland through the network of the Zhongshan Station and China Unicom, completing remote transmission of the entire data set. The communication strategy adopted by the system is duplex communication to achieve the remote setting, debugging, and maintenance of the device [18].

### 2.3. Embedded Software System

The system’s software is an independently developed embedded operating system that has the advantage of less code and more independence of hardware. The data acquisition and transmission of the entire platform and the normal operation of each part are controlled by the operating system. This part of the work has been used in the separately developed polar-region environmental parameter-monitoring system and the Huailai test-field pixel-scale validation monitoring network [16]. After the on-site operation, application, and validation, a large amount of data has been obtained.

## 3. Observation Strategies

### 3.1. GPS Positioning Principle

There are two main types of GPS positioning methods: absolute positioning (also known as point positioning) and relative positioning (also known as differential GPS).

#### 3.1.1. Absolute Position

**Figure 2:**
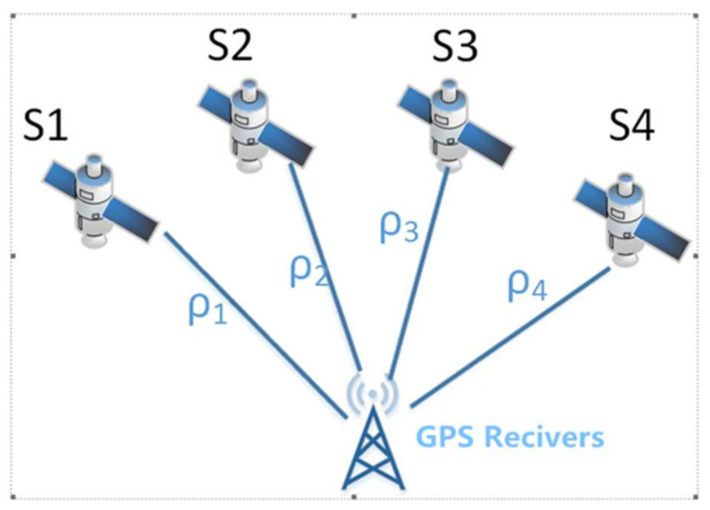
GPS single-point positioning schematic.

The basic principle of GPS absolute positioning is to determine the position of the user’s receiver antenna based on known satellite instantaneous coordinates, benchmarked against the distance observations between the GPS satellite and the user’s receiver antenna. The essence of this positioning method is spatial distance resection, so only three independent distance observations (ρ1, ρ2, ρ3) are needed in one station. However, since GPS adopts the principle of one-way measurement, it is difficult to maintain strict synchronization between the satellite clock and the receiver clock. Because the distance between the actually observed satellite and the station is affected by the synchronization difference between the receiver and the satellite clock, it is also called pseudo distance measurement. The clock difference caused by the satellite clock can be corrected by the corresponding clock-difference parameter provided in the satellite navigation message, but the clock difference caused by the receiver is generally hard to accurately measure in advance. As a result, in the process of data processing, the clock difference can be solved with the coordinates of the observation site as one of the unknown parameters, δt. To solve four unknown parameters (three coordinate quantities and one clock-difference parameter) for one station, there should be at least four synchronous pseudo-range observations, which means it is necessary to observe more than four satellites simultaneously [19], as shown in Figure 2.

#### 3.1.2. Relative Positioning

**Figure 3:**
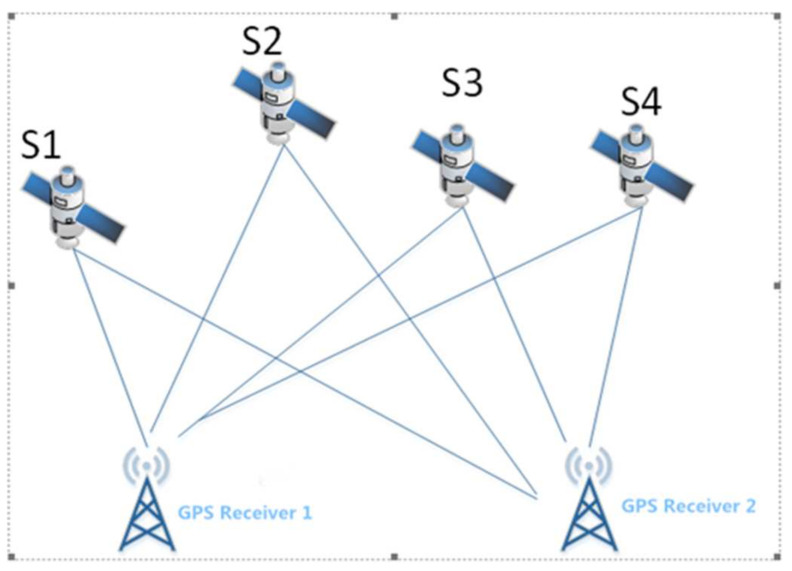
GPS relative positioning principle.

The positioning accuracy in GPS single-point positioning is affected by factors such as clock errors, signal propagation errors, and satellite-orbit errors. Although some of the system errors can be reduced by model correction, the corrected residual errors are still not negligible. Therefore, GPS relative positioning, also called GPS differential positioning, has been developed to effectively minimize these errors. Similarly, GPS relative positioning is also divided into static relative positioning and dynamic relative positioning. The basic schematic diagram is shown in Figure 3.

The basic concept of static relative positioning is to place receivers at the two endpoints of the baseline separately and to keep their positions stationary, while simultaneously observing the same four or more identical satellites to determine the relative positions of the two ends of the baseline in the protocol earth-coordinate system. Under normal circumstances, the accuracy of the broadcast ephemeris can reach approximately 10-7-10-6. If precision ephemeris and orbit-improvement technology are adopted, the positioning accuracy can be improved to approximately 10-9-10-8. It is impossible for other detection methods to achieve such positioning accuracy in mobile monitoring.

The basic concept of dynamic relative positioning is that according to the correlation of the GPS measurement error, the method of relative-positioning operation can be adopted in GPS dynamic positioning, namely GPS dynamic relative positioning. Code-based pseudo-range dynamic relative positioning can achieve a positioning accuracy up to the meter level or sub-meter level when the distance between the base station and the user’s motion station is less than 100 km. Moreover, the positioning accuracy of the carrier phase dynamic relative positioning can reach approximately 1–2 cm in a small area range (less than 30 km).

### 3.2. Selection of System Observation Strategies

The error in GPS point positioning is generally at the level of a meter, which is not enough to meet the accuracy requirements of ice monitoring of the Antarctic ice shelf. Moreover, the GPS monitoring system does not move much with the ice shelf. Thus, GPS static relative positioning to monitor the ice shelf is pre-selected for the system. The monitoring control network consists of the monitoring equipment that is planned to be installed at three different monitoring points and Zhongshan Station, which is used as the observation station, for monitoring the movement of the control points [20].

In GPS static relative positioning, the greatest impact on the positioning accuracy is the satellite ephemeris error. There are two main sources of satellite ephemeris: broadcast ephemeris and precise ephemeris. The broadcast ephemeris is the main data carried in the satellite telegram and can be directly observed and downloaded by the GPS receiver. It is an ephemeris launched by the US GPS Control Center using satellite tracking stations. However, due to current technology limitations, we cannot fully grasp the influence of various perturbation factors on the satellites. Therefore, the broadcast ephemeris data have a larger error. In ordinary point positioning, the error in accuracy of using the broadcast ephemeris to observe and locate is generally approximately 5–10 m, which is a relatively large error. The precision ephemeris is an ephemeris obtained by real-time tracking of the measured data of the satellite at a precisely known observation point. As the precise ephemeris does not need to be extrapolated, it has higher precision. However, the acquisition of precision ephemeris generally takes one to two weeks, which indicates that its real-time capability of positioning is relatively poor. Therefore, to monitor the movement of the ice shelf in real time, the broadcast ephemeris will be used directly to solve for the positioning coordinates.

The static relative positioning method is adopted for mobile monitoring of the Antarctic ice shelf. The satellite ephemeris error between two adjacent stations has a strong correlation, so the phase observation difference measurement method is used to lessen the impact of the broadcast ephemeris error. The influence of the ephemeris error on the positioning is usually estimated by the following formula:

(1) $$\frac{d\rho }{\rho }\approx \frac{dD}{D}\;or\;\frac{d\rho }{\rho }\leq \frac{dD}{D}\leq \frac{1}{4}\cdot \frac{d\rho }{\rho }$$

where ρ is the geometric distance between the satellite and the station; dρ is the ephemeris error; *D* is the baseline vector length; and *dD* is the baseline measurement error caused by the ephemeris. If the baseline length *D* = 20 km, satellite height ρ = 2000 km, and ephemeris error dρ = 25 m, then 2.5 mm ≤ *dD* ≤ 6 mm. The method of static relative positioning can effectively lessen the influence of the satellite ephemeris error on GPS positioning [21,22].

## 4. System Test

After the system prototype was completed, system tests were carried out, especially for GPS data positioning accuracy tests and equipment power-supply systems that require special attention in real-time observations in Antarctica. In the equipment operation test, other functional tests are normal. The abnormal situation was improved, and the abnormality test after the improvement was carried out. In order to obtain the real operating conditions of the equipment, all test data are retrieved from the storage of the mainboard data SD card.

**Figure 4:**
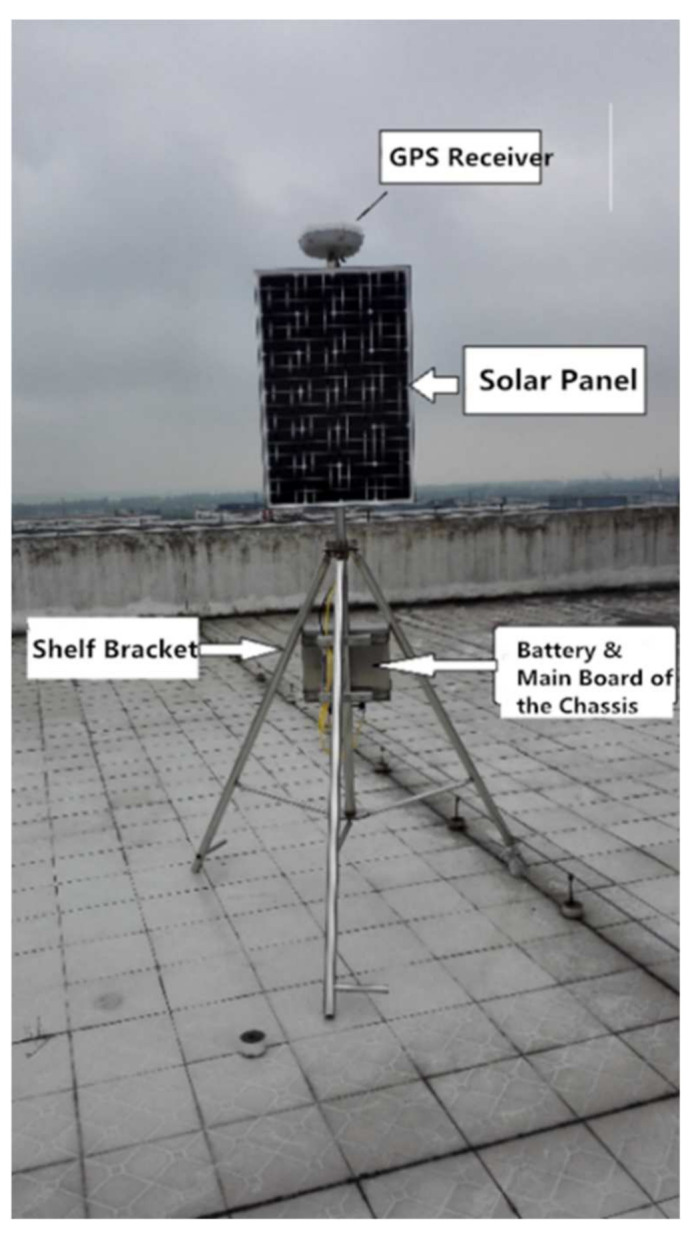
The test physical object.

Due to the special environment of the Antarctic, with snow all over the ground, the entire monitoring platform was placed on a special tripod. When making observations in Antarctica, the special tripod should be fixed with steel wire; the case should be buried in the snow as much as possible so that it can play a role in heat preservation. The monitoring platform of the test physical object is shown in Figure 4.

The selection of GPS test points should follow these principles. First, the field of view should be wide, and there should be no obstacles above 15° around the field of view to prevent GPS signals from being blocked. Second, the test points should be located away from high-power radio sources and high-voltage transmission lines to avoid interference from electromagnetic signals on the GPS signals. Third, there should not be a large area of water near the test point to reduce the impact of multipath effects on GPS measurements. Following these test principles, the test equipment was placed on top of a 9-story building, thereby fully meeting the test principles [23,24].

### 4.1. Test of the GPS Receiver Positioning Measurement Error

The main tests of GPS positioning of the system were the static point positioning test and the mobile monitoring accuracy test by static relative positioning. Since there was no GPS base station at the test site, the precise coordinates of the station could not be accurately located. Therefore, the relative position change of the baseline vector was used in the static relative positioning method to determine the mobile monitoring accuracy of the device.

#### 4.1.1. Ordinary Point Positioning and Precise Point Positioning

In this test, a single system was used for point-positioning testing to confirm the correctness of GPS data acquisition and storage and the measurement accuracy of the GPS receiver. In ordinary point positioning, the positioning error was greater than the meter level. However, with the development of GPS technology, the current method of precise point positioning could greatly improve the accuracy of point positioning.

Online precise point positioning (PPP) is a very popular method for obtaining high-precision point-positioning coordinates after monitoring. Precise point positioning refers to the positioning solution method using the pseudo-distance and phase collected by a single GPS receiver to solve the satellite orbit and satellite clock error with the observation data obtained by the GPS tracking station established on the ground. This method replaces the satellite clock difference in the solution with known data of the precise ephemeris as the starting point and the satellite clock error obtained by the special method. At present, the most well-known free online PPP solutions are APPS (Automatic Precise Positioning Service), OPUS (Online Positioning User Service), CSRS-PPP (The Canadian Spatial Reference System Precise Point Positioning Service), AUSPOS (The Australian Surveying and Land Information Group’s Online GPS Processing Service.), SCOUT (Scripps Coordinate Update Tool), magic GNSS and GAPS. The online PPP solution method is relatively simple to use, that is, by directly uploading the original observation-data file to the website and having the solution results sent to the user by means of email afterward. The GPS positioning test of this equipment will use the free Canadian online PPP solution (CSRS-PPP) to solve the observation data.

1. Test of Ordinary Point Positioning

**Figure 5:**
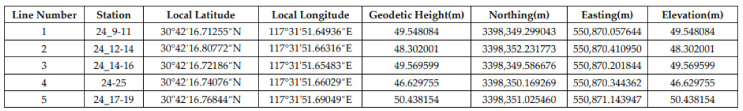
Single-point positioning solution coordinates.

**Figure 6:**
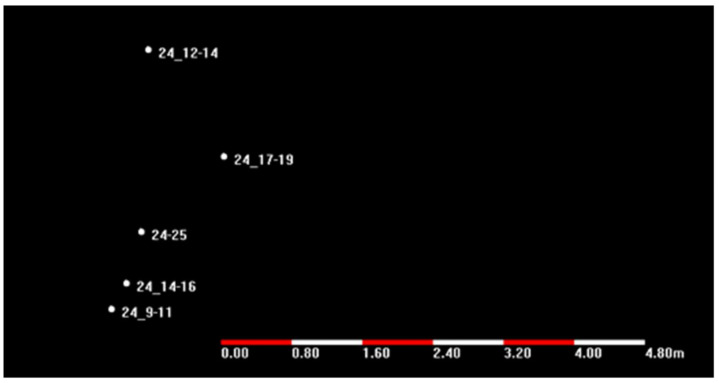
Single-point positioning relative position.

The monitoring device was fixed at one point. Then, the GPS receiver was turned on during different time periods to receive the satellite signal and to obtain the original observation data. After that, the original data of the different time periods were solved by the ordinary point positioning solution and the online precise point positioning solution. To ensure that a static data solution could obtain more real-time solutions, the time of a single measurement was more than two hours. The data were solved using the China GNSS data post-processing software CGO (CHC Geomatics Office). The static point-positioning coordinates were obtained after the observation data of different time segments in the same station were imported into the CGO solution, as shown in Figure 5 and Figure 6.

The test results show that after the point-positioning observation test of the same device at the same point, the system could work normally during the test, and no abnormalities, such as data loss, occurred. In terms of positioning accuracy, the relative coordinate error of the test during different time intervals using the same station was, at most, 4 m, which is relatively low for the use of point positioning. Because many factors, such as satellite ephemeris errors, clock errors, errors caused by ionospheric and tropospheric signal propagation, and errors caused by the tide, will have a relatively large impact on the positioning coordinates during single-point positioning, the positioning-monitoring stability of the system should meet the design requirements. However, it also proves that the accuracy of ordinary point positioning cannot meet the monitoring accuracy requirements of the monitoring of the Antarctic ice shelf and ice-sheet movement.

2. PPP (precision point positioning)

In this test, the single observation time for the same station was six hours. Because the solution coordinates could be obtained after a one-day delay through the online precision-point positioning data solution and the solution process was so unstable that an unsuccessful solution after the data were uploaded often occurred, only the coordinates of the data solution obtained for two different time periods were tested.

**Figure 7:**
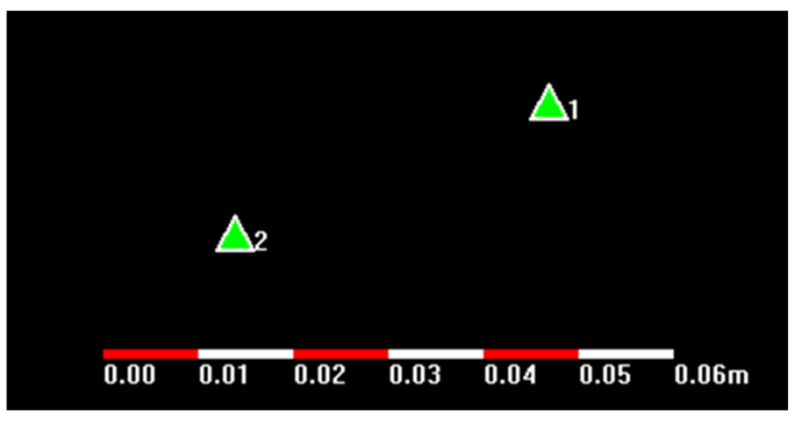
Relative position of two coordinates.

The relative positions of the two coordinates are shown in Figure 7. According to the calculation, the difference between the two coordinate distances was only 3.5 cm. It can be inferred that precise point positioning greatly reduced the positioning error. However, the real-time performance of the precise point positioning was too poor because the solution time delay was long, and a long period of observation was required to obtain coordinates with a relatively satisfactory precision. In the unattended monitoring of the Antarctic ice-shelf movement, it was very difficult to generate energy for the equipment, so this method is not suitable for monitoring in Antarctica.

#### 4.1.2. Test of the System-Monitoring Error

**Figure 8:**
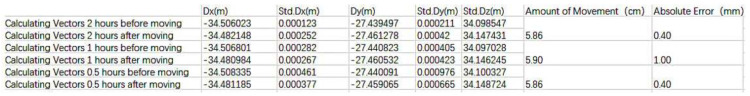
Mobile test results.

The test method was as follows: first, the same period was observed using two devices, A and B, before moving. In addition, the relative baseline vector was then obtained between the two points after the baseline solution. After that, the device was fixed, device B was moved, and the baseline vector of the two points after moving was obtained with the same method. Finally, using the fixed device, A, as the starting point, the displacement distance between the two vectors was calculated to measure the amount of equipment movement. As the movement amount of device B was known in advance through measurement, the absolute measurement error of the movement amount of the GPS could be obtained by comparing the data calculated by the device with known data. The data processing was performed using CGO software and broadcast ephemeris. The data processing took two hours, one hour, or half an hour. The actual movement amount was 5.8 cm, and the test result is shown in Figure 8.

According to the test results, the accuracy of the mobile monitoring measurement by a single reference was very high. In this test, due to the limitations caused by the test conditions, the geometric measurement of the actual movement amount of the system had errors; it was difficult to achieve sub-millimeter accuracy. The absolute error was large. In theory, when the baseline is short, the accuracy of the measurement calculated by the precision ephemeris can reach the sub-millimeter level, while the measurement accuracy of a baseline longer than 20 km can even reach the millimeter level. Luckily, the millimeter-level error of this test fully met the measurement accuracy requirements for the movement-amount determination of the Antarctic ice shelf and ice sheet.

As for the selection of the time of a single simultaneous measurement, it can be seen from Figure 8 that the absolute error difference at half an hour, one hour, and two hours is at the sub-millimeter level because the baseline of this test was shorter. Moreover, the standard deviation of the baseline vectors (Std.Dx, Std.Dy, and Std.Dz) in Figure 8 shows that the standard deviation was not too large when the observation time exceeded 1 h. However, when the observation time was half an hour, the difference between the baseline vectors was larger, and there was relatively less reliability of accuracy. In conclusion, to ensure measurement accuracy and minimize energy consumption of the device at the South Pole at the same time, it is recommended to set the time of simultaneous observation of the device at approximately half an hour to one hour.

### 4.2. Test of the Power-Management System

The system was designed to perform unattended monitoring tasks in the Antarctic. The existence of the Antarctic polar night phenomenon makes the power supply of the equipment a difficult problem to solve. This system used a 12 V 40 Ah low-temperature lithium-ion battery and a 60 W solar panel to provide the energy supply for the system. Undoubtedly, testing the system power supply was also indispensable to ensure the normal operation of the system in the Antarctic.

The test contained two parts. First, during the test of solar-panel charging, the system was put into the outdoors all day, with all tasks open. After one month of continuous testing, the voltage of the system dropped by, at most only, 0.5 V after one night of operation. The battery voltage fluctuated to a peak of 12.32 V per day because the system was designed for low power consumption, and the maximum power consumption of the GPS receiver was only 3 W when it was turned on. In other words, the test results show that when the system was placed in the South Pole, the time for simultaneous observation of the equipment could be set in the daytime so that it could work normally with the solar energy supply.

Because of the existence of the polar night, it was necessary to test the total battery capacity to ensure that the equipment could work normally without solar energy. To slow the test time, the test used a 50 W LED (light-emitting diode) to consume the power of the battery. The test results show that the discharge time could reach approximately 10 h, which implies that the battery power was approximately 500 Wh. Therefore, for equipment with a working energy consumption of only 3 W, if it worked for one hour a day, the continuous working time could reach approximately 160 days. At Zhongshan Station, a full night lasts approximately 60 days, so the system could fully operate normally with its lithium battery during the night.

### 4.3. Test of Other Features

In the equipment operation test, other functional tests are normal. Improvements were made to several abnormal conditions, and a post-condition abnormality test was carried out. The abnormal test mainly tests several conditions, such as low battery power, restoration of power after an abnormal power failure, and acceleration of equipment data-sampling frequency.

The test results indicate that after the system GPS was turned on and the power was restored after abnormal power failure, the system would restart normal work at the time of the next GPS power-on setting. Analysis of the data from the SD card did not reveal any data errors. Additionally, when the system was working normally, there was no error while directly cutting off the power supply and extracting the SD card to parse the data. The only error in this test was that the system’s time would be delayed, which would affect the timing service, resulting in an inconsistent system time of the different systems and affecting the GPS synchronization measurement time. Thus, in the following corrections, the time of the system should be set as the GPS time. The GPS time is very accurate, so the synchronous observation time between different devices can be completely ensured.

The battery voltage was detected by ADC (analog-to-digital converter) when the system was designed. When the battery voltage was lower than a certain condition, the system would automatically enter the sleep state until the battery voltage returned to normal, and the device would resume normal operation without data loss.

After accelerating the system-acquisition frequency and setting the sampling interval of the system battery voltage acquisition at 10 s, even if GPS was turned on all day, the system worked normally without data loss.

The test of abnormality proves that the system could automatically resume normal operation after many non-hardware abnormalities occurred, and it could be fully qualified for unattended monitoring tasks in Antarctica.

### 4.4. Operation in Antarctic Regions

During the Antarctic summer in 2012, when the Chinese Antarctic expedition conducted scientific investigations on the Amery Ice Shelf, 23 consecutive days of GPS observations were conducted at the camp, from 12 February 2012 to 5 March 2012 (local time). The daily observation time was from 07:11 to 07:30 at local time, lasting 19 min.

**Figure 9:**
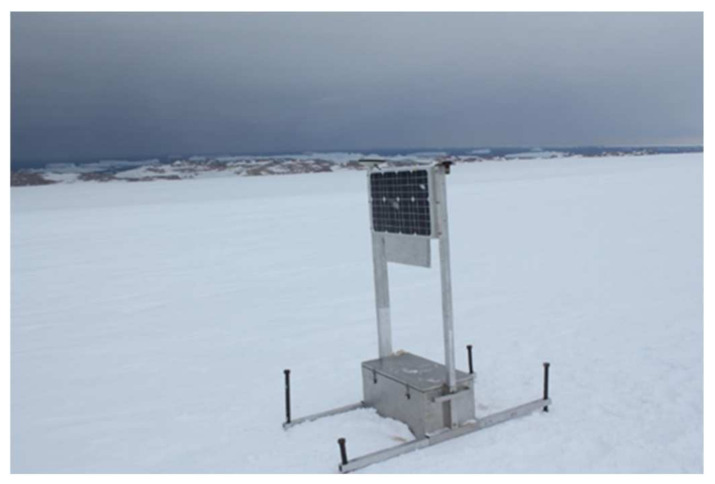
The installation site in Antarctica.

The observation environment of the Amery ice shelf is special. In summer, due to the sun’s irradiation, the surface snow of the ice shelf melts. As a result, the tripod on which the instrument is placed sinks by 0.1 to 0.2 m per day. During data collection, in order to ensure the stability of the tripod in the observation process, certain measures must be taken to reduce influences of the surface snow of the ice shelf on the tripod when the snow is melted by the sun during the day and the environment cools down at night. Therefore, before setting up the tripod, the expedition team stepped on the surface of the snow at the positions where the three legs of the tripod were planned to be placed and smashed three half-meter wooden sticks to support the three legs of the tripod. Before observing, the GPS receiving antenna was installed on the tripod for a while first, so as to stabilize the melting and freezing status of the snow and ice around the stick. After that, the instrument for observation was placed to ensure that the tripod was basically stable during the observation and would not sink. The installation site is shown in Figure 9.

**Figure 10:**
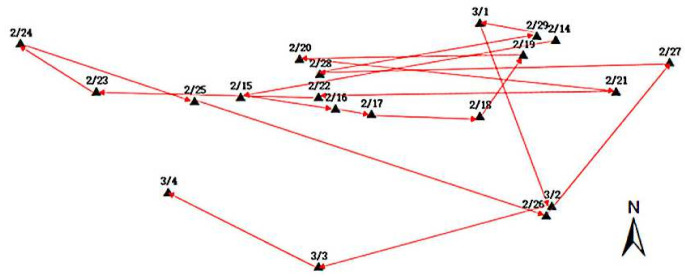
Changes in ice-shelf position.

CGO software was adopted to process the Antarctic GPS monitoring data and the obtained ice-shelf-motion measurement results. The GPS monitoring data of the ice shelf were imported into ArcGIS (a geographic information system platform), and the position change is shown in Figure 10. It can be seen from Figure 10 that the position of the ice shelf was changing every day, and the daily moving distance varied. From the small scale, the movement direction of the ice shelf did not occur strictly in a certain direction. During the observation period, the minimum moving distance was 0.1395 m on 22 February 2012, the maximum moving distance was 10.433 m on 16 February 2012, and the average daily moving distance was 3.702 m.

**Figure 11:**
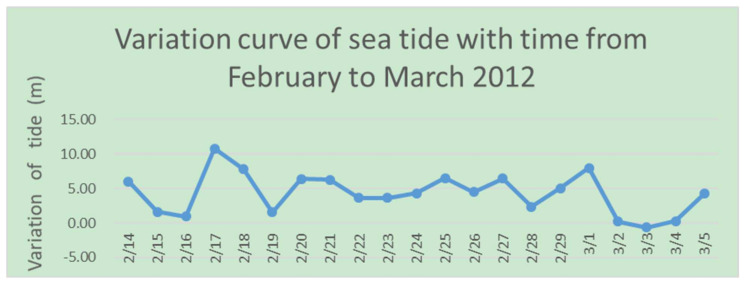
Altitude change of ice shelf.

Through 23 days of continuous GPS monitoring, the time series of the vertical movement of the ice shelf caused by the sea tide was obtained. The altitude change of the ice shelf is shown in Figure 11.

## 5. Remote Sensing Validation

**Figure 12:**
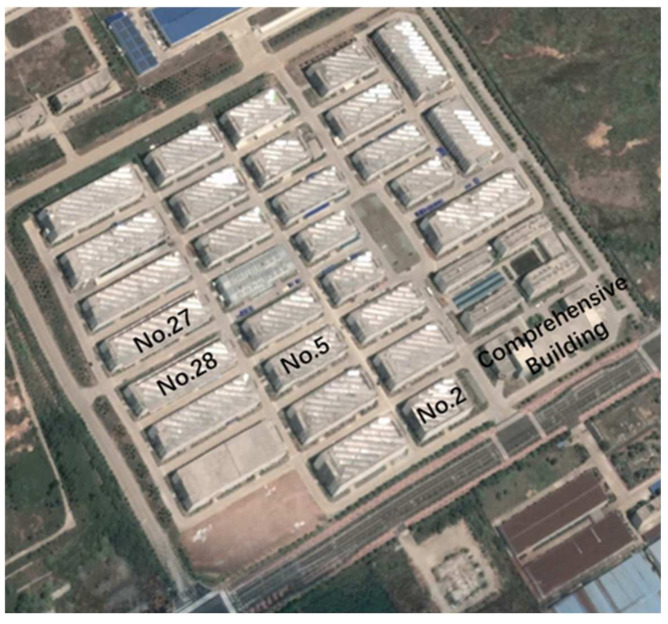
Map of experimental park.

Using the improved devices, a glacier movement simulation experiment was carried out in Chizhou City, Anhui Province, China. The test site was the Electronics Industrial Park in the Economic Development Zone of Chizhou City, where there are many factories. Two improved GPS monitoring devices were installed on the roof of the factory building. The park is shown in Figure 12, a picture taken on Google Maps to show the distribution of different test points in the test area.

**Figure 13:**
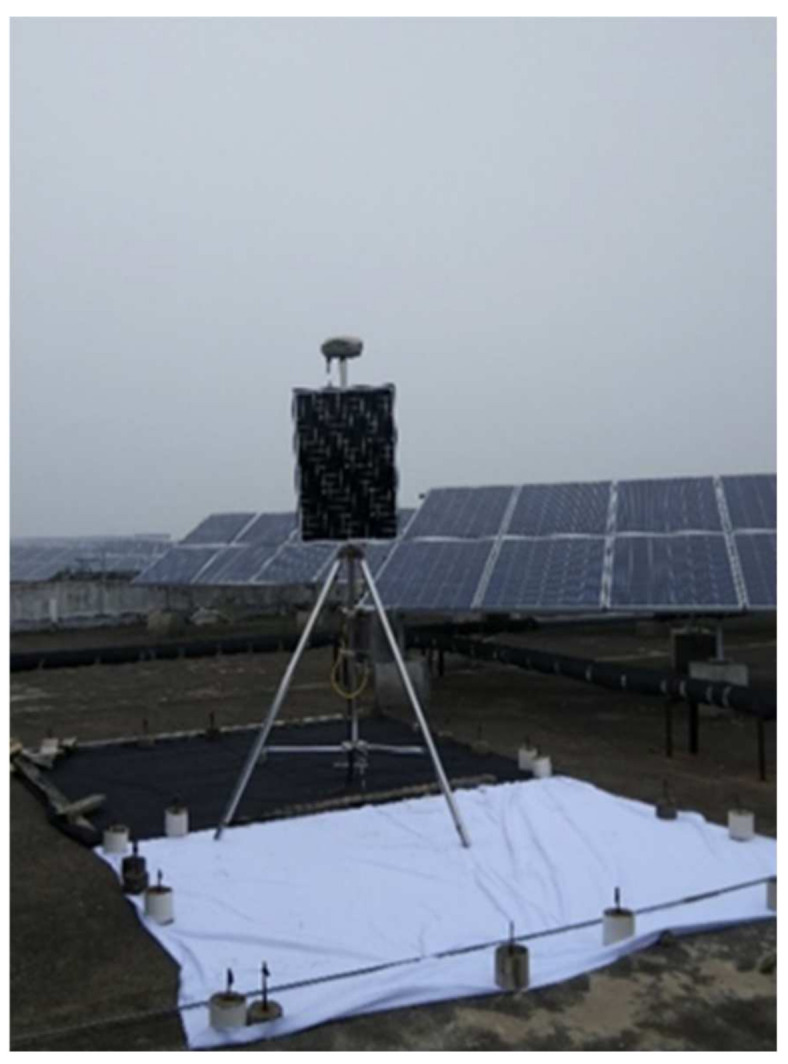
Equipment site installation drawing.

Considering the validation of the later and multi-source satellite remote sensing data, a cloth of nearly 5 m × 5 m (area) was laid under the GPS equipment to improve the recognition of the equipment on the image. The on-site equipment installation is shown in Figure 13.

**Figure 14:**
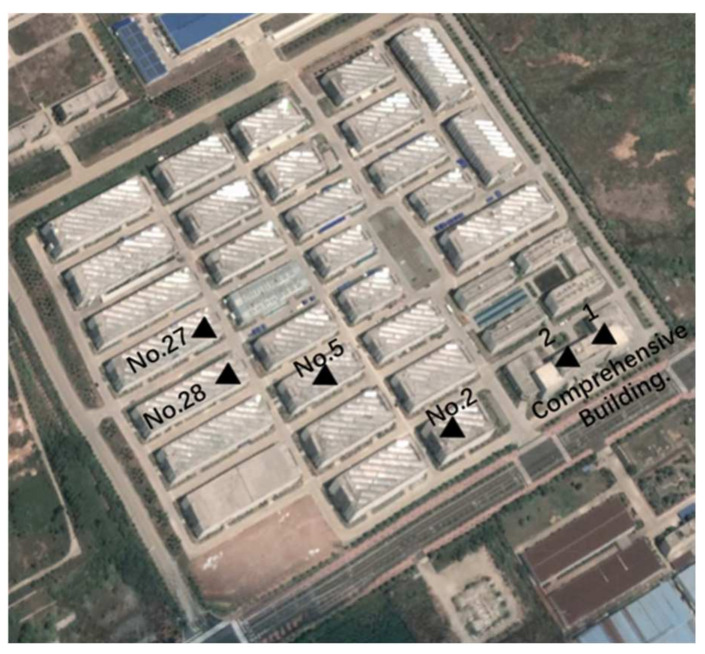
GPS device installation point map.

Initially, the simulation experiment was to set up equipment in the No. 5 building of the industrial park and the No. 1 point of the comprehensive building. After monitoring for some time, the two devices were moved to the No. 28 building and the No. 2 point of the comprehensive building separately to simulate glacier movement. Similarly, after monitoring for some time, they were moved to No. 27 building and No. 2 building. Every time the device moved, drone images and high-resolution satellite remote sensing images were acquired. The point movement of GPS monitoring equipment is shown in Figure 14.

### 5.1. GPS Monitoring Data

**Figure 15:**
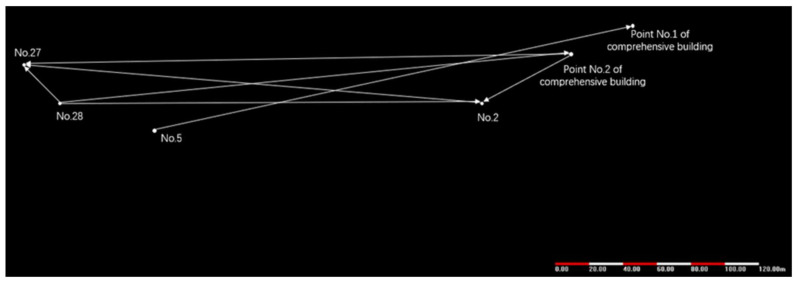
The interface diagram of original data import by CGO.

The GPS monitoring O file was imported into the CGO processing software to obtain the information on each station and process all the baselines. The original data import software interface is shown in Figure 15.

The quality index RMS is set to 40 mm, the horizontal accuracy index is 20 mm, and the vertical accuracy index is 40 mm. From the above baseline calculation, it can be seen that both the baseline calculation result and the control net are qualified.

We used CGO to calculate GPS monitoring data. The solution results are shown in Table 1.

According to the calculation, we obtained the coordinate position of the GPS monitoring device and the displacement of each device. Since high-resolution images cannot obtain elevation information, for the convenience of comparison, the horizontal movement distance of the equipment was calculated, and the comprehensive movement distance of the two pieces of equipment during the experiment was calculated, as shown in Table 2.

### 5.2. High-Resolution Remote Sensing Image

In order to realize the combination of GPS monitoring data and multi-source satellite remote sensing data to improve the accuracy and reliability of satellite remote sensing inversion, we obtained remote sensing images covering the simulated experimental area. The information in Table 3 is as follows. The results of processing six high-resolution images are shown in Table 4.

According to the high-resolution images, we obtained the plane coordinates of the GPS device and the total moving distance of the two devices during the experiment, as shown below in Table 5.

In the simulation experiment, the plane movement distance of GPS device 1 is 110.664 m, while the movement distance in the ZY301_nad and ZY301_mux images are 103.829 m and 130.129 m, respectively. The plane movement distance of GPS device 2 is 103.024 m, while the movement distances in the ZY301_nad and ZY301_mux images are 143.523 m and 112.665 m, respectively. It can be seen that the average error of the ZY301_nad image is 23 m, and the average error of the ZY301_mux image is 14 m.

For comparison of man-made regular mobile monitoring equipment with ZY-3 high-resolution remote sensing images in the same period in the area, the validation conclusion is: The projection area of GPS equipment (≈1 m^2^) is lower than the ground area corresponding to a single pixel of most high-resolution images; The positioning deviation of the device on the image is related to the spatial resolution; Among them, the image deviation of the NAD sensor is about 7–28 m (2–8 pixels); The deviation of the MUX sensor is about 10–25 m (2–5 pixels), and the deviation value is also affected by the image shooting angle.

## 6. Results Analysis and Expectation

### 6.1. Result Analysis

During the operation in the Antarctic, the data were returned via Iridium in real time, costing nearly 20,000 RMB a month, so the observation was ceased. Subsequently, the data were sent back using a message from the China Unicom communication tower at Zhongshan Station in China. A new version was tested during the period from 1 August 2017 to 1 September 2018. In the case of outdoor open air and heavy thunderstorms, the unattended continuous operation test was conducted. The equipment was operating in good condition, and no hardware problems were found. Through the test of GPS positioning accuracy, it was found that the equipment has poor accuracy in single-point positioning. The common single-point positioning error is above the meter level, and the precision single-point positioning error is above the centimeter level. The accuracy of using two devices to differentially measure the amount of movement is at the millimeter level, which is in line with the theoretical GPS measurement accuracy. Thus, the performance test results of the GPS receiver are good, and it can be used in the monitoring of the ice-sheet movement of the Antarctic ice shelf. In the early stage of system operation, the SD card data could not be parsed. After debugging, it was found that the program parameters were incorrectly set. When the problem of data storage and analysis was solved, no data loss occurred during the subsequent testing process. Therefore, the data collected by the device were tested as reliable. In the polar region, there is extreme night weather, so energy is the most important factor. The test results of the power-management system show that the device can completely rely on lithium batteries to complete the one-hour collection frequency. The test results of the equipment operating under abnormal conditions also indicate that the equipment is competent to complete the unmanned ice-shelf motion-monitoring task in the Antarctic. 

In Antarctic and domestic experiments, it can be seen that at least two devices are required to achieve the accuracy of differential measurement of movement to reach the millimeter level. With the increase in monitoring points, it is possible to analyze the changes in motion parameters, such as the three-dimensional motion speed of the ice shelf and sea tide difference at different positions, which is beneficial for deepening our understanding of ice-shelf motion. In addition, the more GPS monitoring points, the smaller the data error of the joint solution will be, and more parameter information can also be solved.

### 6.2. Expectation

Due to limiting conditions, the experiment is still being carried out intermittently. Although the previous experiments were short, the obtained polar ice-shelf motion data sets with continuousness, high sampling frequency, high accuracy, and long-term sequence have made it possible to study the motion of the ice-shelf system on a small time scale. The horizontal movement trend, speed, and regular changes in the elevation of the ice shelf during the observation period were obtained. The tide-change parameters of the ocean tide at the observation point were preliminarily derived and can be compared with the previous research data of the Amery ice-shelf movement in the future. The GPS precision single-point positioning technology used by the system can also solve the ice flow velocity, ice flow direction and elevation change at the ice-shelf observation points. Moreover, analysis of the change in the ocean tide at the observation points can provide a basis for subsequent material-balance calculation.

In addition to real-time and long-term monitoring of the ground, real-time ground monitoring can also verify remote sensing products, thereby improving the accuracy of remote sensing product inversion. Ground monitoring is point data, and remote sensing is surface data. More work is needed to study the fusion of the two types of data; it is a big challenge to send data back in real time or quasi-real time in the Antarctic region. The existing communication costs are relatively high.

There are plans to build an integrated air-space-ground monitoring system in the tri-polar region, including the Antarctic, the Arctic, and the Qinghai-Tibet Plateau. The system is a monitoring network that integrates ground-sensor networks, remote sensing, and drones and can realize unattended, all-weather continuous observations.

## Figures and Tables

**Table 1:** GPS solution result table.

Type	Date	12/9–3/4	4/18–5/19	5/20–6/25
GPS 1	Position	No.5	No.28	No.27
	X (m)	550,640.5126	550,586.556837	550,608.6943
	Y (m)	3,398,309.747929	3,398,329.452	3,398,415.739
	Z (m)	38.596945	41.746395	43.822881
GPS 2	Position	Point No.1 comprehensive building	Point No.2 comprehensive building	No.2
	X (m)	550,920.570819	550,883.0502	550,830.829
	Y (m)	3,398,379.45	3,398,363.768	3,398,328.851
	Z (m)	61.131541	62.039906	52.321517

**Table 2:** GPS equipment monitors displacement changes.

Type	GPS 1		GPS 2	
	Actual Moving Distance (m)	Horizontal Movement Distance (m)	Actual Moving Distance (m)	Horizontal Movement Distance (m)
First move	57.5273 m	57.4411 m	40.66783 m	40.6660 m
Second move	89.106 m	89.0815 m	63.376 m	62.8192 m
Comprehensiveness	110.7873 m	110.6639 m	103.3996 m	103.0236 m

**Table 3:** List of high-score images of the study area.

Number	Date	Satellites and Equipped Sensors	Spatial Resolution	Product Grade
1	19 December 2017	ZY-3 (bwd)	2.1 m	Firsts
2	19 December 2017	ZY-3 (fwd)	3.5 m	Firsts
3	19 December 2017	ZY-3 (mux)	6 m	Firsts
4	19 December 2017	ZY-3 (nad)	3.5 m	Firsts
5	19 December 2017	ZY-3 (mux)	6 m	Firsts
6	19 December 2017	ZY-3 (nad)	3.5 m	Firsts

**Table 4:** High-resolution image-processing results.

Image type	Date	Position	North Coordinate	East Coordinate
ZY301_nad	19 December 2017	Point No.1 comprehensive building	13,083,539.197	3,418,049.719
		No.5	13,083,249.059	3,418,045.213
ZY301_mux	19 December 2017	Point No.1 comprehensive building	13,083,557.026	3,418,089.963
		No.5	13,083,280.491	3,418,054.999
ZY301_fwd	19 December 2017	Point No.1 comprehensive building	13,083,578.217	3,418,181.347
		No.5	13,083,302.476	3,418,146.383
ZY301_bwd	19 December 2017	Point No.1 comprehensive building	13,083,523.977	3,417,896.218
		No.5	13,083,223.338	3,417,859.135
ZY301_nad	11 August 2018	No.2	13,083,442.013	3,417,953.958
		No.27	13,083,133.56	3,418,032.793
ZY301_mux	11 August 2018	No.2	13,083,419.619	3,417,970.007
		No.27	13,083,149.598	3,418,016.889

**Table 5:** The plane movement distance of the GPS device on the high-resolution image.

Image Type	GPS	Plane Moving Distance (m)
ZY301_nad	GPS 1	103.8299298
	GPS 2	143.5227973
ZY301_mux	GPS 1	130.1286193
	GPS 2	112.665006

## Data Availability

The study did not report any data.
